# Anti-Stress and Anti-Depressive Effects of Spinach Extracts on a Chronic Stress-Induced Depression Mouse Model through Lowering Blood Corticosterone and Increasing Brain Glutamate and Glutamine Levels

**DOI:** 10.3390/jcm7110406

**Published:** 2018-10-31

**Authors:** Hyeonwi Son, Soonwoong Jung, Jung Hye Shin, Min Jung Kang, Hyun Joon Kim

**Affiliations:** 1 Bio Anti-Aging Medical Research Center, Department of Anatomy and Convergence Medical Science, Institute of Health Sciences, Gyeongsang National University School of Medicine, Jinju 52727, Korea; hw.son@gnu.ac.kr (H.S.); birth1110@gnu.ac.kr (S.J.); 2 Namhae Garlic Research Institute, Namhae 52430, Korea; whanbee@hanmail.net (J.H.S.); Jung-75@hanmail.net (M.J.K.)

**Keywords:** spinach, chronic stress, corticosterone, depressive behavior

## Abstract

Spinach is one of the most widely consumed vegetables, and is known as for both physical and mental health maintenance. However, there is little information about how spinach protects one from stress. In the present study, we created three extracts from *Spinach oleracea* L., (frozen powder (FP), water extract (WE), and ethanol extract (EE)), and examined their anti-stress and anti-depressive effects on mouse using a chronic immobilization stress (CIS) regimen. FP, WE, and EE showed different free amino acid constituents. Calorie-balanced diets derived from each extract were tested for their ability to reduce blood corticosterone (CORT) levels in naïve mice. Diets supplemented with FP or EE induced lower blood CORT levels than a normal diet, but the WE diet did not. Mobility duration and sucrose preference were increased by FP and EE supplementation in the CIS-induced depression animal models. Moreover, FP and EE increased glutamate and glutamine levels in the medial prefrontal cortex (mPFC) compared with CIS-induced depressed group. These results suggest that spinach has anti-stress and anti-depressive properties by lowering CORT and increasing glutamate and glutamine levels in the mPFC.

## 1. Introduction

Stress challenges the homeostasis of animals and requires adaptive responses. Animals adapt to various stressors by the endogenous stress response system initiated through the hypothalamus pituitary adrenal (HPA) axis, leading to increased serum corticosteroid levels. However, the prolonged and excessive elevation of stress or stress hormone levels is deleterious to the animal [1]. Many modern lifestyles expose humans to diverse and consistent stressors that cause various diseases, including major depressive disorder (MDD), a devastating psychiatric illness that induces disability and sometimes leads to suicide. The World Health Organization predicted that depression would be the second leading cause of disability by 2020 in 2001, but in fact, by 2013 MDD was already the second leading cause of disability [2]. Thus, finding a safe and effective anti-depressive alternative medicine for regulating daily stress hormone levels is crucial. Currently available antidepressants are monoaminergic and have been prescribed to MDD patients following their serendipitous discovery in the 1950s. Over the next three decades, persistent research efforts have improved monoaminergic antidepressants as selective agents (e.g., selective serotonin reuptake inhibitors (SSRIs)) and patient quality of life. Nevertheless, the clinical limitations of these compounds still exist [3]. For example, MDD patients must wait weeks or months to benefit from the antidepressant’s efficacy, and one-third of patients do not respond to the medication. Up to 70% of recovered MDD patients experience minor residual symptoms and some major recurring symptoms. Additionally, patients taking antidepressants often suffer side effects such as nausea, insomnia, weight gain or loss, decreased sexual drive, drowsiness, fatigue, and headaches [4]. These limitations demonstrate the need to find new anti-depressant targets and drugs for treating MDD.

A high concentration of glucocorticoids is one cause of MDD [5], according to preclinical studies. Chronic glucocorticoid treatment changes cellular functions and structures in the brain and induces depressive behaviors [6,7]. Cellular atrophy induced by glucocorticoids is similar to neuronal atrophy found in depressed patients [8]. Moreover, many studies have shown that successful antidepressant therapies are associated with normalization of impairments in negative feedback of the HPA axis [9]. Therefore, elevated glucocorticoids are a key neurobiological factor in eliciting depressive behaviors, suggesting that maintaining homeostatic levels of glucocorticoids may be an effective therapeutic strategy against MDD.

In a previous study, we reported that exogenous glutamine (Gln) directly infused into the medial prefrontal cortex (mPFC) attenuates depressive-like behaviors of mice [10]. We recently reported that Gln-supplemented diet reduced blood corticosterone (CORT) levels and maintained normal growth performance of cage-reared chicks [11]. Moreover, the Gln-supplemented diet ameliorated the deleterious effects of the chronic immobilization stress (CIS)-induced depression mouse model [12]. In that study, we found that the Gln-supplemented diet reversed their depressive behaviors and restored brain glutamate (Glu) and Gln levels. It is well-known that spinach contains many amino acids, including Gln, and other beneficial compounds, such as minerals, essential vitamins, folic acid, lecithin, secretin, saponins, and flavonoids [13]. Spinach’s possible beneficial effects on stress and mood are also of interest, but no scientific research regarding this issue has been published. Therefore, we investigated if spinach extracts attenuate blood CORT levels and depressive-like behaviors induced by CIS.

## 2. Materials and Methods

### 2.1. Animals

Male C57BL/6 mice (Koatech, Co. Ltd., Pyeongtaek, Korea) weighing 22–24 g at the start of the experiment were used for all studies. All animals were single-caged in a temperature- and humidity- controlled room (lights on 06:00–18:00) with food and water available ad libitum. Mice were randomly grouped according to body weight using a computer-generated list. Animal use procedures were performed in accordance with National Institutes of Health guidelines and an approved protocol (GLA-100917-M0093) by the Gyeongsang National University Institutional Animal Care & Use Committee.

### 2.2. Spinach Extracts

Spinach (*Spinach oleracea* L.) was purchased from Namhae County, Republic of Korea. After cutting roots, spinach was washed, lyophilized, and powdered to generate a frozen powder (FP). Separately, 10 L water or ethanol were added to 500 g powdered spinach, and the mixture was normalized to room temperature for 24 h and filtered (No. 2 filter paper, Advantec, Tokyo, Japan). The water extract (WE) was generated through lyophilizing, and the ethanol extract (EE) was concentrated using a rotary evaporator (N-1200 AVW, EYELA, Tokyo, Japan).

### 2.3. Free Amino Acids Analysis of Spinach Extracts

To determine free amino acid content, 150 mL ethanol were added to 3 g spinach extract, and the mixture was homogenized and centrifuged (5000 rpm for 10 min). The residue was extracted twice with 75 mL 80% ethanol, and the supernatant was collected, concentrated, and defatted with ether to a final volume of 50 mL. The mixture was concentrated using a rotary vacuum evaporator and adjusted to 10 mL with lithium citrate buffer (pH 2.2). The sample was filtered through a 0.25 µm-membrane. We identified the amino acid peaks in the samples by their retention times compared with external standards. Amino acid concentrations were quantified according to the relative peak height measured (Biochrom 30+, Biochrom Ltd., Cambridge, UK) [14,15].

### 2.4. Free Glu and Gln Analysis in the PFC

L-Glu and L-Gln contents were determined by ultra-performance liquid chromatography (UPLC) with an AccQ-Tag Ultra system (Waters, Milford, MA, USA) as previously reported [16]. The brain tissues were homogenized in T-PER (tissue protein extraction reagent) (Pierce, Rockford, IL, USA) and the supernatant was collected after centrifugation at 12,000 rpm for 30 min. Protein concentration in the supernatant was determined using the Bicinchoninic Acid (BCA) reagent (Pierce, Rockford, IL, USA). The sample was ultra-filtered using a SmarPor Syringe Filter (25 mm, 0.2 µm, Woongki Ltd., Seoul, Korea). Once the filtrate was diluted to a suitable concentration, fluorescence derivatization was performed according to the AccQ-Tag manufacturer’s instruction. A 20-µL sample solution, 60 µL of AccQ-fluor borate buffer, and 20 µL of AccQ-fluor reagent were mixed together, and the mixture was incubated for 5 min at 55 °C. Derivatized amino acids were separated on an AccQ-Tag Ultra column (2.1 × 100 mm, Waters) by gradient elution (AccQ-Tag Ultra eluent A and B) at 30 °C. The derivatized amino acids were detected by a PDA eλ detector (Waters) [17,18]. The chromatography data were analyzed using Empower software (Waters). To determine amino acid concentrations, a standard solution containing known concentrations of amino acids were analyzed with samples in every series.

### 2.5. CIS Regimen

The regimen was carried out as previously described [19,20]. Briefly, mice were repeatedly placed in a restrainer 2 h/day for 15 days under 100 lx light. Body weight and food intake were evaluated every other day throughout the experiment. Mice were fed a diet containing one of the spinach extracts (FP (80 g), WE (40 g), and EE (34 g)) per one kilogram of feed or a normal diet for two weeks, and total calories fed were adjusted to 4000 kcal/kg (AIN 93G, UniFaith, Seoul, Korea).

### 2.6. Behavioral Tests

The tail suspension test (TST) was conducted as previously described [12,21] with some modification. Mice were individually suspended by the tail via a horizontal bar approximately 30 cm from the floor using tape approximately 1 cm from the tip of the tail. The 6 min motion was recorded and analyzed with an animal behavior video program (EthoVision, Noldus Information Technology, Wageningen, The Netherlands).

The sucrose preference test (SPT) was performed to determine symptoms of anhedonia as previously described [20] with some modification. Briefly, mice were habituated for 48 h with a palatable sucrose solution (0.1 M), followed by a 24 h water deprivation period and 6 h access to two identical bottles, one filled with sucrose solution and the other with water. The consumption of sucrose solution and water was measured throughout the 6 h period, and sucrose preference was represented as the ratio of sucrose-to-water consumption.

### 2.7. Measurement of Plasma CORT

Plasma CORT was measured as previously described [22]. Mouse blood was collected at 09:00 into vacutainers containing K3 EDTA. Plasma was isolated via centrifugation at 1000× *g* for 15 min at 4 °C. The samples were stored at −80 °C until the assay was performed. Quantitation of plasma CORT levels was carried out using the CORT EIA kit (Cayman Chemical, Ann Arbor, MI, USA) according to the manufacturer’s protocol.

### 2.8. Statistical Analyses

All data evaluated by ANOVA and a Newman Keuls multiple comparison *post-hoc* test using Prism (GraphPad Software, La Jolla, CA, USA). Data are represented as means ± SEM. The cut-off for statistical significance was *p* < 0.05.

## 3. Results

### 3.1. Free Amino Acid Contents of Spinach Extracts Differ by the Extraction Method

Total free amino acids in the WE were 5.56 times higher than the FP extract (Table 1). Interestingly, Gln was detected in the EE (29.13 mg/100 g) and FP (62.88 mg/100 g), but not in the WE. Instead of Gln, WE contained a high level of Glu (1171.38 mg/100 g) compared with FP (146.69 mg/100 g) and EE (23.65 mg/100 g). Conversely, L-tyrosine was not detected from WE, but was found in EE and FP at 162.88 and 38.75 mg/100 g, respectively.

### 3.2. FP and EE-Supplemented Diets Decrease Blood CORT Levels in Mice

**Figure 1:**
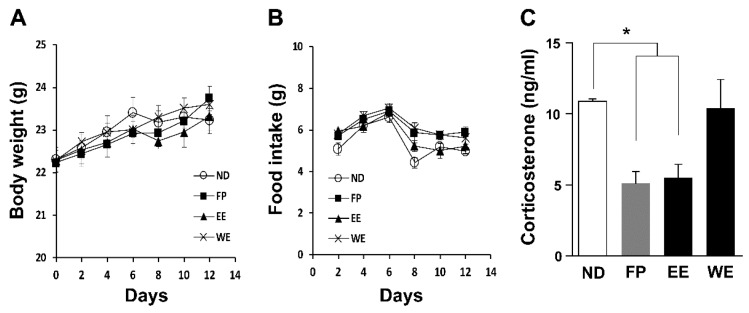
A spinach extract diet reduced blood CORT level. (**A**) Body weight and (**B**) food intake (*n* = 8 mice per group). (**C**) Blood CORT decreased in mice fed an FP and EE diet but not a WE diet (*n* = 5 mice per group). All values are means ± SEM. * *p* < 0.05. EE, ethanol extract; FP, frozen powder; ND, normal diet; WE, water extract; CORT, corticosterone.

We observed no changes in body weight and food intake between mice fed diets containing different spinach extracts (Figure 1A,B), it would likely to be due to the calorie-balanced diet prepared for this experiment. Because we recently found that Gln-supplemented diet decreased blood CORT level in growing chicks [11] and CIS-induced depressive mice [12], we expected that Gln-containing extract would be effective on reduction of blood CORT level. Expectedly, blood CORT levels were remarkably decreased by EE- and FP-supplemented diets (Figure 1C) although all groups suffered from same daily stressors. This result suggested that FP and EE could have anti-stress and anti-depressive properties; thus we used EE and FP for further analyses but did not use WE.

### 3.3. FP and EE-Supplemented Diet Attenuates Depressive-Like Behaviors Induced by CIS

**Figure 2:**
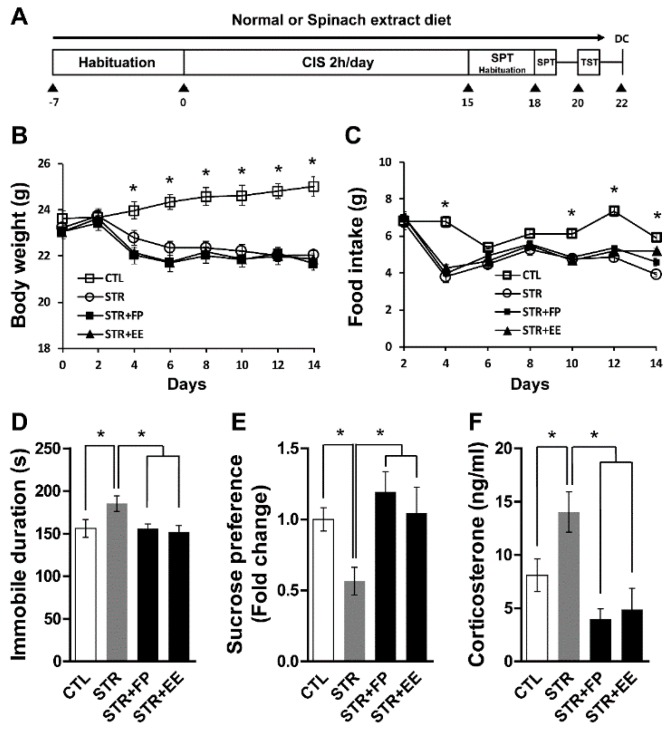
Spinach extract diet attenuated depressive behaviors and increased CORT in a CIS-induced depression model. (**A**) Timeline of CIS experiments. (**B**) Body weight and (**C**) food intake (*n* = 7–8 mice per group). (**D**) Immobile duration and (**E**) sucrose preference, assessed by the TST and SPT, respectively (n = 7–8 mice per group). (**F**) Blood CORT level (*n* = 4–5 mice per group). All values are means ± standard error of mean (SEM). * *p* < 0.05. CTL, control; DC, decapitation; EE, ethanol extract; FP, frozen powder; ND, normal diet; STR, stress; CIS, chronic immobilization stress; SPT, sucrose preference test; TST, tail suspension test.

We determined if spinach extracts have anti-depressive effects on mice exposed to CIS. Mice were fed with normal or FP- or EE-supplemented diets during the experiments depicted in Figure 2A. The body weight and food intake significantly decreased in the stress groups (STR, STR + FP, STR + EE) compared with that of the control group (CTL) (Figure 2B,C) were found. Significantly decreased sucrose preference and increased immobility were seen in mice given CIS compared with the CTL. These depressive-like behaviors were reversed by FP and EE supplementation compared with the STR (Figure 2D,E), highlighting the antidepressant properties of these particular spinach extracts. We also found that FP and EE significantly attenuated increased blood CORT levels by CIS (Figure 2F). These results demonstrate that spinach extract-supplemented diets decrease stress-associated blood CORT levels and ameliorate depressive-like behaviors induced by chronic stress.

### 3.4. FP and EE-Supplemented Diet Increases Brain Glu and Gln Levels

**Figure 3:**
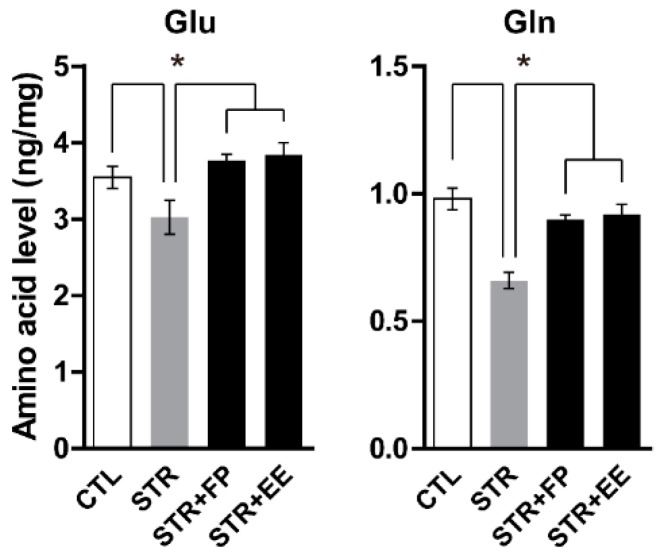
Glu and Gln changed by CIS and spinach extract-supplemented diet during chronic immobilization stress. Glu and Gln levels in the prefrontal cortex (PFC; *n* = 5–6 mice per group). All values are means ± standard error of mean (SEM). * *p* < 0.05. CTL, control; EE, ethanol extract; FP, frozen powder; STR, stress.

Previously, we demonstrated that increased level of Gln by direct infusion into the mPFC has an anti-depressant effect on chemically induced depressive mice [10] and recently found the causal relationship between low levels of cortical Glu and Gln and depressive behaviors of stressed mice [12]. Moreover, we showed that Gln-supplemented diet has antidepressant effects through increments of brain Glu and Gln levels [12]. Therefore, we examined the possibility the FP- and EE-supplemented diets could change brain Glu and Gln levels and found that there were remarkable increments of Glu and Gln levels in the PFC in the FP- and EE-supplemented groups compared with the normal diet STR group (Figure 3).

## 4. Discussion

In the present study, we found that FP and EE from spinach decreased blood CORT levels in human-handled mice. We also showed that FP and EE reversed blood CORT levels and depressive behaviors induced by CIS. Moreover, FP and EE increased Glu and Gln levels compared with the STR group. Thus, it is suggested that the FP and EE spinach extracts have anti-stress and anti-depressant properties against chronic stress.

Natural products from foods can be good candidates for alternative medical therapies and have attracted the attention of many researchers and pharmaceutical companies because their bioactivities are comparably effective and safe compared with chemical drugs. Numerous natural product-derived medicines have been developed for various diseases [23]. Only St John’s wort, an herbaceous perennial plant commonly found in Asia and Europe, has been licensed and widely prescribed to MDD patients in many European countries. Recent meta-analyses suggest that St John’s wort is safer than and comparably efficacious with SSRIs in patients with MDD [24]. As a result, many studies have examined natural extracts’ effects on depressive-like behaviors using animal models [25,26,27].

Spinach is called a “superfood” because of its constituents and is traditionally used as a functional food for various purposes. The functionality of spinach has been confirmed with respect to its anti-anemia, anti-microbial, anti-convulsant, anti-diabetic, anti-hyperlipidemic, anti-inflammatory, anti-oxidant, and anti-ulcer activities [28], suggesting the potential anti-stress property of spinach extracts in a supplemented diet. Moreover, the vitamins, folic acid, and flavonoids present in spinach exert anti-depressive properties [29,30,31,32]. Our results support the anti-stress and anti-depressive activities of spinach being alluded by previous studies.

Depending on the extraction method, the constituents of amino acids from different organisms vary [33,34,35]. We used three extraction methods in our current study, and although only free amino acids were analyzed, the extracts’ contents were significantly different depending on the extraction method. Only FP and EE of spinach reduced CORT levels, further demonstrating that beneficial properties of a natural product may depend on the extraction method. From this result, we hypothesize that the absence of L-Gln and L-tyrosine in the WE spinach may explain its lack of effect on blood CORT levels in the mice. People take L-tyrosine supplements for improving depression, attention-deficit/hyperactivity disorder, cognitive performance, narcolepsy, and alertness following sleep deprivation [36]. Tyrosine positively affects the activity of catecholamines, including epinephrine and norepinephrine, which regulate stress responses in the adrenal glands and brain [37]. Thus, tyrosine can confer benefits under conditions such as stress, cold, fatigue, and prolonged sleep deprivation [38].

In addition, Gln is an important nitrogen and carbon source for many cell types [39] and may promote gut function, immune responses, and other essential physiological processes during times of stress, such as physical exhaustion and post-operative periods [40]. Gln is also essential for maintaining the homeostasis of Glu, which is involved in brain neurotransmission [10]. Previously, we demonstrated Gln deficiency in prefrontal cortical neurons could evoke depressive-like behaviors in rodents [10] and low levels of Glu and Gln by CIS would be a cause for depressive behaviors [12]. There were several lines of evidence showing low levels of Glu and Gln in certain brain regions of MDD patients [41,42,43]. In this study, we also found the lower levels of Glu and Gln in the PFC of the STR group (Figure 3) which is consistent with our previous reports [10,12]. Interestingly, FP and EE increased the levels of Glu and Gln in the PFC (Figure 3), which may activate glutamatergic neurons being suppressed by CIS [12]. These findings suggest that the beneficial effects of spinach FP and EE on stress hormones and depressive-like behaviors may be due to decreasing blood CORT and increasing Glu and Gln levels in the PFC.

Although we confirmed FP and EE of spinach relieve stress-related and depressive-like symptoms induced by CIS, we only analyzed their free amino acid contents. Spinach contains other beneficial compounds, including minerals, vitamins A, B, C, D, E, and K, folic acid, lecithin, secretin; saponins and flavonoids [13]. Thus, further analyses of FP, EE, and WE content are necessary to identify other candidates that may reduce stress and depressive-like behaviors.

## Figures and Tables

**Table 1:** Contents of free amino acids in spinach extracts (mg/100 g).

Amino Acids	Ethanol Extract	Water Extract	Frozen Powder
L-Phenylalanine	ND	44.13 ± 0.21	1.48 ± 0.15
L-Aspartic Acid	26.11 ± 1.31	ND	ND
L-Threonine	32.28 ± 1.54	68.44 ± 1.36	11.18 ± 0.45
L-Serine	32.11 ± 1.36	98.80 ± 6.32	23.78 ± 0.36
L-Asparagine	23.68 ± 0.25	3.43 ± 0.24	30.55 ± 0.33
L-Glutamate	23.65 ± 0.84	1171.38 ± 15.27	146.69 ± 2.58
L-Glutamine	29.13 ± 0.25	ND	62.88 ± 3.36
L-Proline	549.98 ± 5.64	564.45 ± 4.25	145.72 ± 4.68
Glycine	9.24 ± 0.24	66.79 ± 0.65	5.45 ± 0.65
L-Alanine	62.88 ± 2.65	233.74 ± 3.54	20.23 ± 0.36
L-Citrulline	13.98 ± 0.25	44.80 ± 3.54	2.54 ± 0.04
α-Aminobutyric acid	8.74 ± 0.21	17.03 ± 0.25	0.65 ± 0.15
L-Valine	80.82 ± 0.14	105.14 ± 4.62	24.23 ± 1.23
L-Cystine	2.36 ± 0.73	0.35 ± 0.03	2.46 ± 0.54
L-Methionine	11.19 ± 0.21	58.20 ± 1.75	16.39 ± 0.36
L-Isoleucine	104.12 ± 0.41	168.15 ± 4.56	40.07 ± 0.45
L-Leucine	43.77 ± 0.36	94.14 ± 2.36	17.09 ± 0.50
L-Tyrosine	162.88 ± 10.94	ND	38.75 ± 0.61
β-Alanine	29.41 ± 0.21	79.61 ± 1.25	14.30 ± 0.45
L-Homocystine	189.05 ± 8.50	668.55 ± 4.66	83.38 ± 0.86
γ-Amino-n-butyric acid	158.99 ± 7.64	231.14 ± 1.35	42.32 ± 0.64
Ethanolamine	108.29 ± 3.50	448.94 ± 4.25	20.58 ± 0.03
δ-Hydroxylysine	0.10 ± 0.02	89.18 ± 0.58	2.43 ± 0.05
Ornithine	5.48 ± 0.14	75.49 ± 0.25	1.73 ± 0.04
1-Methylhistidine	8.96 ± 0.65	43.87 ± 0.36	13.21 ± 0.10
L-Histidine	9.47 ± 1.58	26.47 ± 1.27	2.77 ± 0.05
L-Arginine	16.84 ± 0.24	0.28 ± 0.04	19.97 ± 1.25
Total	1743.51 ± 49.81	4402.50 ± 62.96	790.83 ± 20.27

ND: not detected.
